# Distribution, carbapenem resistance and DTR dynamics of ESKAPEEc pathogens from pediatric bloodstream infections in Sichuan Province, 2017-2025: epidemiological characteristics under COVID-19 pandemic and emerging linezolid MIC creep

**DOI:** 10.3389/fcimb.2026.1914448

**Published:** 2026-10-01

**Authors:** Shanshan Long, Min Zhong, Xin Liu, Kai Zhang, Zhuorui You, Xiangning Huang, Hua Yu

**Affiliations:** 1 Department of Laboratory Medicine, Sichuan Provincial People’s Hospital, University of Electronic Science and Technology of China, Chengdu, China; 2 School of Medicine, University of Electronic Science and Technology of China, Chengdu, China

**Keywords:** bloodstream infection, carbapenem resistance, COVID-19, difficult-to-treat resistance (DTR), ESKAPEEc, MIC drift, pediatrics, Sichuan province

## Abstract

**Objective:**

To characterize the distribution and antimicrobial resistance evolution of ESKAPEEc pathogens causing pediatric bloodstream infections in Sichuan Province across the COVID-19 era, and to systematically evaluate the epidemiology of carbapenem resistance (CR), difficult-to-treat resistance (DTR), and MIC drift in vancomycin and linezolid, thereby addressing a long-standing gap in longitudinal pediatric multidrug-resistant surveillance in Southwest China.

**Methods:**

A multicenter retrospective study was conducted using pediatric blood culture data from 134 healthcare institutions in Sichuan Province between 2017 and 2025. A total of 20,018 isolates were included and stratified into pre-pandemic, pandemic, and post-pandemic periods. Antimicrobial susceptibility testing and DTR classification were interpreted according to CLSI M100 (35th edition) criteria. Statistical analyses were performed using WHONET 5.6 and SPSS 26.0.

**Results:**

A total of 5,236 ESKAPEEc isolates were identified, accounting for 26.2% of all bloodstream isolates. Detection rates increased during the pandemic period and declined thereafter. Carbapenem resistance rates in *Escherichia coli* and *Klebsiella pneumoniae* were significantly higher in the post-pandemic period compared with the pre-pandemic baseline. The overall DTR prevalence was 8.6%, with *Acinetobacter baumannii* exhibiting the highest DTR burden (87.3%) despite a declining temporal trend, whereas DTR in *K. pneumoniae* increased progressively over time. Reliance on CR criteria alone failed to identify a substantial proportion of DTR-positive *E. coli* isolates. All *Staphylococcus aureus* isolates remained susceptible to vancomycin and linezolid *in vitro*, with stable vancomycin MIC distributions; however, a high-MIC subpopulation among linezolid-treated MRSA isolates increased significantly over time (r=0.767, *P* = 0.016). Distinct age-stratified differences were observed in both pathogen distribution and resistance profiles.

**Conclusions:**

The COVID-19 pandemic exerted a bidirectional and sustained impact on the pediatric ESKAPEEc landscape and resistance ecology in Sichuan Province. Post-pandemic escalation of carbapenem resistance persists. The persistently high DTR burden in A. baumannii and early MIC drift toward linezolid in MRSA represent two critical regional threats in pediatric infectious disease management. Regional surveillance systems should incorporate DTR metrics alongside CR monitoring and implement age-stratified, precision-based antimicrobial stewardship strategies.

## 1 Introduction

Bloodstream infection (BSI) is a major contributor to severe pediatric hospitalization, clinical deterioration, and mortality, with neonates and younger children experiencing a higher infection risk and poorer outcomes (Lindell et al., 2020; Kritsotakis et al., 2022). In recent years, the incidence and mortality of pediatric BSI have remained persistently high worldwide, and the emergence of multidrug-resistant organisms has further substantially complicated clinical antimicrobial therapy (Folgori et al., 2014; Kern and Rieg, 2020; Yang et al., 2023). The ESKAPEEc group represents the most critical multidrug-resistant pathogens implicated in healthcare-associated bloodstream infections, encompassing the *Enterococcus faecium, Staphylococcus aureus, Klebsiella pneumoniae, Acinetobacter baumannii, Pseudomonas aeruginosa, Enterobacter* spp.,and *Escherichia coli*. These organisms account for 50%–70% of bloodstream isolates worldwide, with marked heterogeneity in detection rates across countries and regions. Infections due to these pathogens may lead to sepsis and septic shock, and are strongly correlated with adverse outcomes such as ICU admission, extended hospital stays, and mortality, underscoring their clinical priority for prevention and control (De Angelis et al., 2018; Marturano and Lowery, 2019; Miller and Arias, 2024).

The COVID-19 pandemic has exerted a complex bidirectional influence on bacterial resistance ecology. On one hand, strengthened hospital infection control measures, including isolation policies and enhanced disinfection protocols, have reduced cross-transmission of pathogens. On the other hand, the increased number of critically ill patients and expanded exposure to broad-spectrum and high-grade antimicrobials have continuously selected for multidrug-resistant strains (Rawson et al., 2020; Wee et al., 2021; Yang et al., 2024). However, most existing surveillance studies have been restricted to a single phase of the pandemic, and substantial regional heterogeneity exists, with a lack of longitudinal pediatric data spanning the pre-pandemic, pandemic, and post-pandemic periods (Li et al., 2024; Ahmed et al., 2025; Li et al., 2025).

Conventional classifications of multidrug resistance (MDR) and extensively drug-resistant (XDR) phenotypes are increasingly inadequate to reflect the true clinical challenge of “no effective therapeutic options.” In this context, DTR has emerged as a more clinically relevant classification system in recent years (Kadri et al., 2018; Tamma et al., 2022). In addition, MIC drift in vancomycin and linezolid among Staphylococcus aureus may progressively compromise antimicrobial efficacy and serves as an early warning indicator of resistance evolution in Gram-positive pathogens (Jian et al., 2020; Haas et al., 2023). To date, no longitudinal surveillance integrating DTR classification and linezolid MIC drift in pediatric populations has been conducted in Southwest China, leaving a substantial epidemiological gap.

Sichuan Province, characterized by its vast geographic span and uneven distribution of healthcare resources, exhibits distinct regional patterns of pediatric antimicrobial resistance. Our previous studies covered only partial phases of the COVID-19 period and did not systematically investigate DTR dynamics or MIC evolutionary trends, leaving important gaps in temporal characterization (Long et al., 2025). In this study, leveraging the Sichuan Provincial Antimicrobial Resistance Surveillance Network, we included pediatric blood culture isolates collected from 134 medical institutions across the province over a continuous nine-year period (2017-2025), thereby establishing a large-scale cohort spanning the entire COVID-19 era.

This study aims to comprehensively delineate temporal shifts in pediatric ESKAPEEc distribution, carbapenem resistance, DTR prevalence, and linezolid MIC drift under pandemic influence, and to further elucidate age-stratified differences in resistance patterns. By addressing a critical absence of integrated surveillance data in Southwest China, these findings are expected to provide an evidence-based foundation for stratified antimicrobial therapy and regional resistance control strategies.

## 2 Materials and methods

### 2.1 Study design and data sources

This multicenter retrospective surveillance study was conducted using data obtained from the Sichuan Provincial Bacterial Resistance Surveillance Network, encompassing 134 healthcare institutions across 21 prefecture-level cities in Sichuan Province, including 113 tertiary hospitals and 21 secondary hospitals. Isolates were collected between January 1, 2017 and December 31, 2025. According to the national COVID-19 control phases in China, the study period was stratified into three stages: pre-pandemic (2017-2019), pandemic (2020-2022), and post-pandemic (2023-2025).

### 2.2 Inclusion and exclusion criteria for isolates

Inclusion criteria were as follows: (1) pediatric patients aged ≤14 years; (2) positive blood cultures with clinically confirmed bloodstream infection; (3) isolates identified as ESKAPEEc pathogens (*Enterococcus faecium, Staphylococcus aureus, Klebsiella pneumoniae, Acinetobacter baumannii, Pseudomonas aeruginosa, Enterobacter* spp.*, Escherichia coli*); for coagulase-negative staphylococci, inclusion required concordant positivity in paired blood cultures; (4) only the first isolate was retained when identical organisms were repeatedly isolated from the same patient within 30 days; and (5) complete bacterial identification and antimicrobial susceptibility data were available.

Exclusion criteria included: (1) single-positive coagulase-negative staphylococci isolates; (2) Isolates with a VITEK identification confidence level of <95% or a MALDI-TOF MS score of <2.0 were excluded. Low-confidence identification may lead to species misclassification, thereby introducing bias into the statistical estimates of pathogen spectra and antimicrobial resistance rates; and (3) Specimens with collection, transport, or testing procedures that did not comply with standard protocols—including blood culture contamination, hemolyzed specimens, delayed delivery beyond the recommended time frame, and incorrect container usage—were excluded. Such specimens may compromise the reliability of organism isolation and antimicrobial susceptibility testing results.

### 2.3 Bacterial identification and antimicrobial susceptibility testing

Bacterial identification was performed using the VITEK 2 automated microbial identification system (bioMérieux, Inc., Hazelwood, MO, USA) or MALDI-TOF mass spectrometry(bioMérieux S.A., Marcy-l’Étoile, France). Antimicrobial susceptibility testing was conducted using broth microdilution and the Kirby-Bauer disk diffusion method. Minimum inhibitory concentrations (MICs) for vancomycin and linezolid in *Staphylococcus aureus* were determined using broth microdilution or E-test gradient strips. All susceptibility interpretations strictly followed the Clinical and Laboratory Standards Institute (CLSI) M100, 35th edition (2025) guidelines (Clinical and Laboratory Standards Institute, 2025).

### 2.4 Definitions of antimicrobial resistance and age stratification

#### 2.4.1 ESKAPEEc group

The ESKAPEEc group included: *E. faecium, S. aureus, K. pneumoniae, A. baumannii, P. aeruginosa, Enterobacter* spp., and *E. coli.*

#### 2.4.2 Resistance phenotypes

Carbapenem resistance (CR) was defined as resistance to either imipenem or meropenem. Fluoroquinolone resistance (FQR) was defined as resistance to either ciprofloxacin or levofloxacin. DTR in Gram-negative bacteria was defined as intermediate susceptibility or resistance to all first-line β-lactam agents and fluoroquinolones (Kadri et al., 2018). Methicillin-resistant *Staphylococcus aureus* (MRSA) was defined as an oxacillin MIC ≥4 μg/mL.

#### 2.4.3 Age groups

Patients were stratified into four age groups: neonates (≤28 days), infants (29 days to <1 year), toddlers (1 to <3 years), and children (3 to 14 years).

#### 2.5 Statistical analysis

Statistical analyses were performed using WHONET 5.6 for isolate distribution, resistance rates, and MIC distributions, and SPSS 26.0 for hypothesis testing. Categorical variables were summarized as counts, proportions (%), and resistance rates (%). Comparisons among multiple groups were conducted using the chi-square test or Fisher’s exact test, with *post hoc* pairwise comparisons performed when overall differences were significant (*P* < 0.05). Temporal trends in resistance were assessed using the Cochran-Armitage chi-square trend test (reported as P trend). MIC temporal trends were evaluated using Spearman rank correlation analysis (reported as r and P values). All tests were two-sided, with a significance threshold of α = 0.05 and *P* < 0.05 considered statistically significant.

### 2.6 Ethical approval

This study was approved by the Ethics Committee of Sichuan Academy of Medical Sciences & Sichuan Provincial People’s Hospital (Approval No. 2026467). As all data were retrospectively de-identified and anonymized, informed consent was waived due to the impossibility of patient re-identification.

## 3 Results

### 3.1 Temporal distribution of bloodstream pathogens and ESKAPEEc isolates across pediatric age groups

A total of 20,018 pediatric BSI isolates collected between 2017 and 2025 were included, of which 5,236 (26.2%) were classified as ESKAPEEc pathogens. Across the defined COVID-19 phases, the proportion of ESKAPEEc isolates showed a non-linear trajectory, increasing during the pandemic period and declining thereafter: 25.6% in the pre-pandemic phase, 34.3% during the pandemic, and 21.1% in the post-pandemic phase (**Figure 1**).

**Figure 1:**
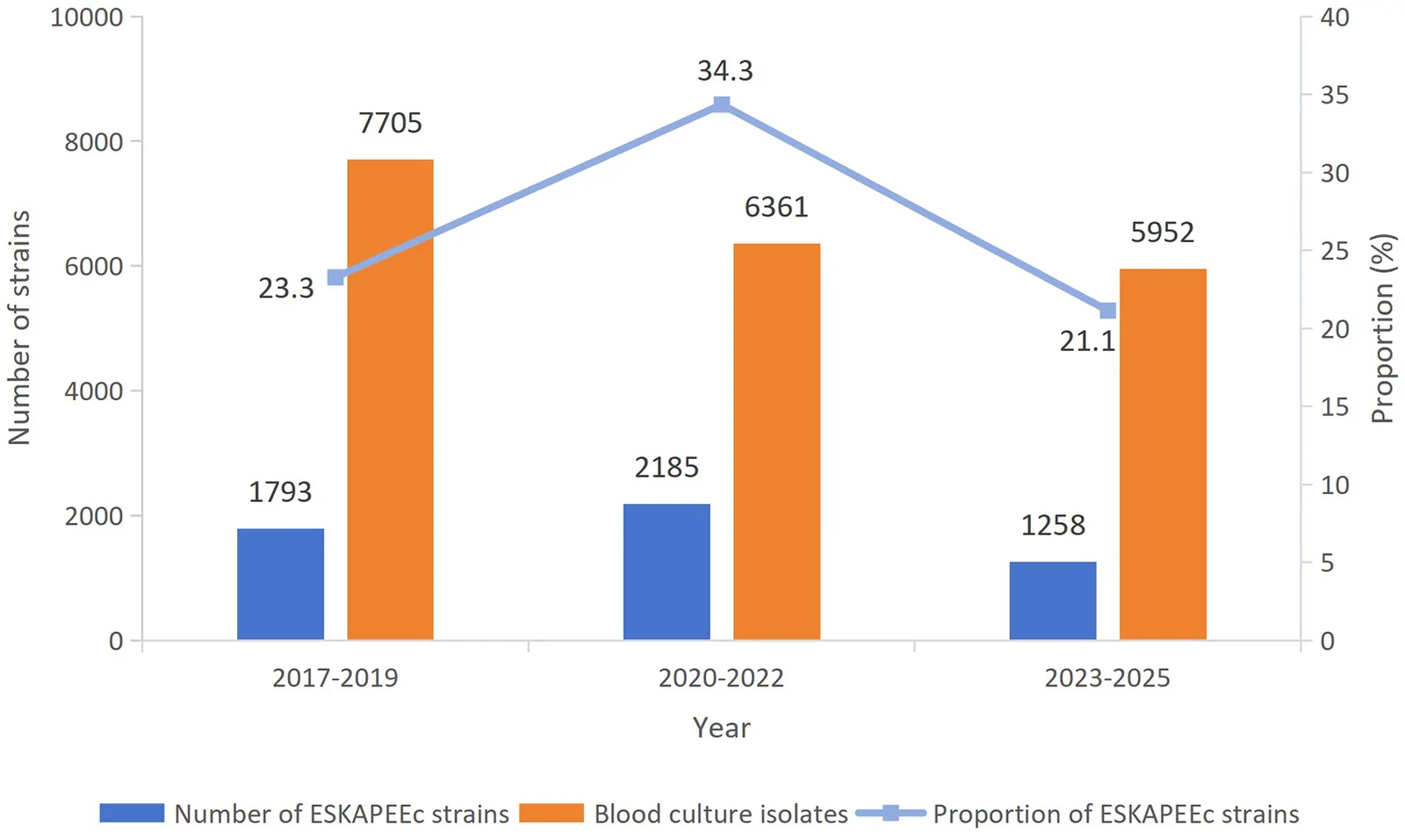
Temporal distribution of ESKAPEEc isolates across three COVID-19 pandemic phases, 2017–2025. Blue bars represent the count of ESKAPEEc strains isolated from blood cultures; orange bars indicate the total number of pediatric blood culture isolates in each period. The light blue line shows the proportion of ESKAPEEc strains among all bloodstream isolates. The three time intervals were divided according to the COVID-19 epidemic stages: 2017–2019 (pre-pandemic), 2020–2022 (pandemic peak), and 2023–2025 (post-pandemic).

Within the ESKAPEEc group, *Escherichia coli* accounted for the largest proportion (40.1%), followed by *Staphylococcus aureus*, *Klebsiella pneumoniae*, *Enterobacter cloacae*, *Pseudomonas aeruginosa*, and *Acinetobacter baumannii* (**Figure 2**).

**Figure 2:**
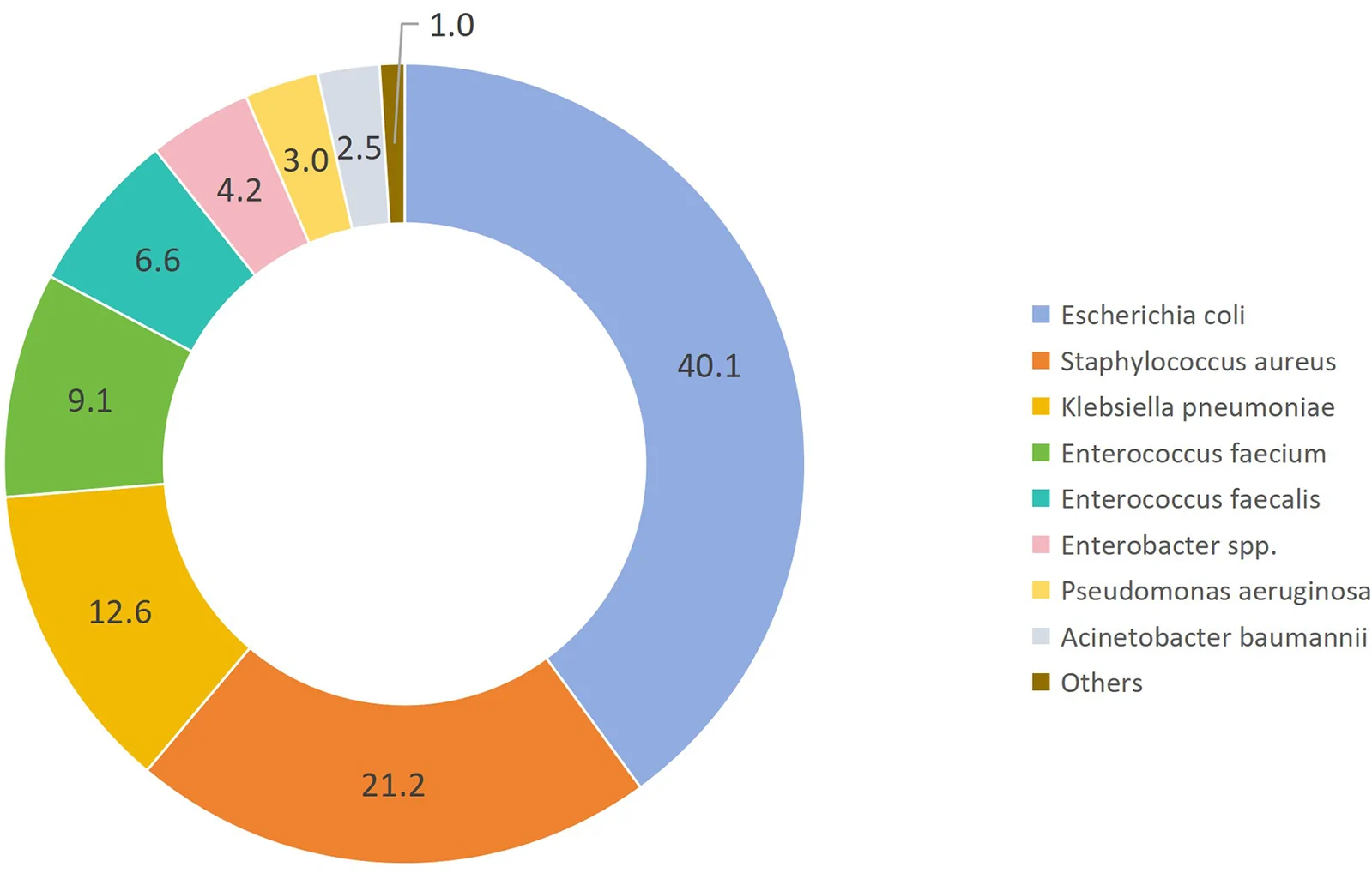
Composition of ESKAPEEc isolates from pediatric bloodstream infections, 2017–2025. Donut chart showing the species distribution of ESKAPEEc pathogens recovered from children with bloodstream infections. Values inside each sector correspond to the percentage of each bacterial genus/species among all ESKAPEEc isolates. The “Others” group covers rare ESKAPEEc species with an overall proportion less than 1%.

Distinct age-dependent distributions of predominant BSI pathogens were observed (**Table 1**). In neonates, infants, and toddlers, coagulase-negative staphylococci predominated among the top-ranked pathogens. In contrast, *Staphylococcus aureus* was the leading pathogen in children aged 3–14 years. Among representative ESKAPEEc organisms, *E. coli* consistently ranked among the top five pathogens across all younger age strata, whereas *K. pneumoniae* entered the top five only in neonates. *S. aureus* was primarily prominent in toddlers and older children. Notably, coagulase-negative staphylococci and *Streptococcus pneumoniae* were not classified within ESKAPEEc and represented common non-target dominant pathogens across all age groups.

**Table 1:** Baseline distribution of top 5 pathogens causing pediatric bloodstream infections in different age subgroups.

Age group	Isolates	Rank 1	Rank 2	Rank 3	Rank 4	Rank 5
Neonates (≤28d)	6,042	Staphylococcus epidermidis*	***Escherichia coli***	Staphylococcus hominis*	Staphylococcus haemolyticus*	***Klebsiella pneumoniae***
Infants (29d–<1y)	6,548	Staphylococcus epidermidis*	Staphylococcus hominis*	***Escherichia coli***	*Streptococcus pneumoniae*^	Staphylococcus haemolyticus*
Toddlers (1y–<3y)	4,174	Staphylococcus epidermidis*	*Streptococcus pneumoniae*^	Staphylococcus hominis*	***Escherichia coli***	***Staphylococcus aureus***
Children (3y–<14 y)	3,254	***Staphylococcus aureus***	Staphylococcus epidermidis*	Staphylococcus hominis*	***Escherichia coli***	*Streptococcus pneumoniae*^

*Coagulase-negative staphylococci (CoNS); ^*Streptococcus pneumoniae* are not classified as ESKAPEEc pathogens. Taxa shown in bold correspond to ESKAPEEc organisms.

### 3.2 Temporal dynamics of antimicrobial resistance in major Gram-negative bacteria across the three COVID-19 phases

Resistance profiles of five major multidrug-resistant Gram-negative organisms demonstrated heterogeneous evolutionary patterns across the pre-pandemic, pandemic, and post-pandemic periods (**Figure 3**). Statistically significant temporal trends in DTR were observed only for *Acinetobacter baumannii* and *Klebsiella pneumoniae* (**Table 2**).

**Figure 3:**
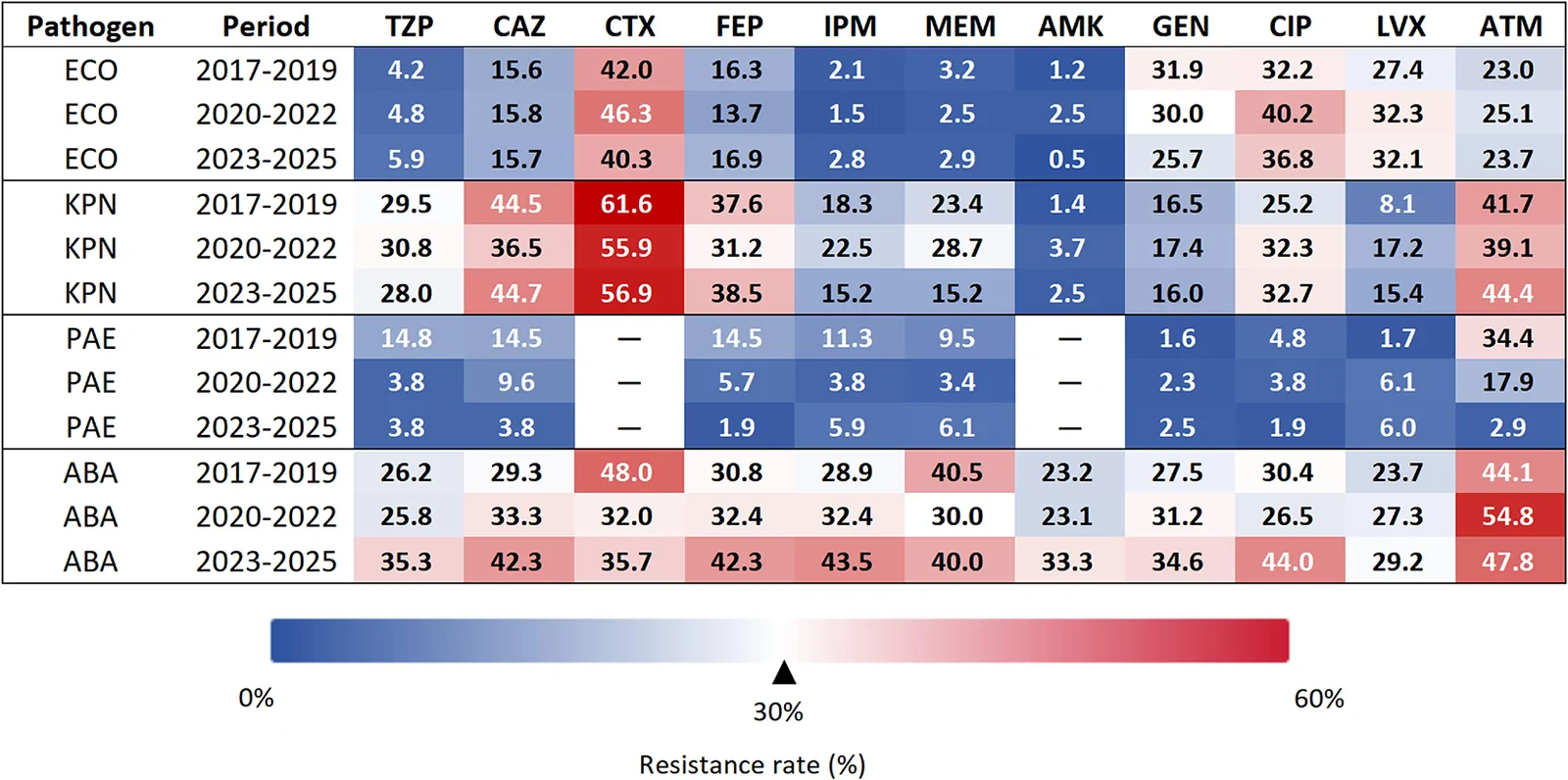
Heatmap of antimicrobial resistance rates of four predominant Gram-negative bloodstream pathogens across three study periods (2017–2025). Color gradient represents resistance rates, with the scale truncated at 60%; dark blue corresponds to 0% resistance, and deep red represents resistance rates ≥60%. ECO, *Escherichia coli*; KPN, *Klebsiella pneumoniae*; PAE, *Pseudomonas aeruginosa*; ABA, *Acinetobacter baumannii*. TZP, piperacillin-tazobactam; CAZ, ceftazidime; CTX, cefotaxime; FEP, cefepime; IPM, imipenem; MEM, meropenem; AMK, amikacin; GEN, gentamicin; CIP, ciprofloxacin; LVX, levofloxacin; ATM, aztreonam. The symbol “−” indicates that susceptibility testing was not conducted for the corresponding pathogen-antibiotic pair.

**Table 2:** Dynamic changes in the prevalence of difficult-to-treat resistance (DTR) among five Gram-negative bacteria across three pandemic periods.

Pathogen	Total	Pre-pandemic period (2017–2019)	Pandemic period (2020–2022)	Post-pandemic period (2023–2025)	*P* for trend
*Acinetobacter baumannii*	87.3 (124/142)	92.6 (75/81)	82.9 (29/35)	76.9 (20/26)	0.04
*Klebsiella pneumoniae*	16.5 (118/714)	9.7 (27/279)	15.0 (37/246)	28.6 (54/189)	<0.001
*Escherichia coli*	1.6 (36/2265)	1.5 (12/814)	1.7 (14/816)	1.6 (10/635)	0.9
*Pseudomonas aeruginosa*	8.4 (14/167)	6.5 (4/62)	9.4 (5/53)	9.6 (5/52)	0.3
*Enterobacter cloacae*	4.6 (10/219)	2.3 (2/86)	8.1 (5/62)	4.2 (3/71)	0.51

P for trend was calculated using the Cochran-Armitage trend chi-square test to assess linear trends in DTR detection rates across successive pandemic stages (pre-pandemic, pandemic, post-pandemic).

The DTR positivity rate of *A. baumannii* showed a sustained linear decline from the pre-pandemic to the post-pandemic period (Ptrend = 0.040). In contrast, the DTR detection rate in *K. pneumoniae* increased significantly and linearly over time (Ptrend < 0.001). No significant temporal trends in DTR prevalence were observed for *Escherichia coli*, *Pseudomonas aeruginosa*, or *Enterobacter cloacae* (all *P* trend > 0.05).

Further correlation analyses confirmed a strong association between the DTR phenotype and carbapenem resistance (**Table 3**). Across all pandemic phases, carbapenem non-susceptibility rates were consistently and significantly higher in DTR-positive isolates than in DTR-negative isolates, regardless of species.

**Table 3:** Cross-analysis of the correlation between DTR phenotype and carbapenem resistance.

Pathogen	Phenotype	CR-positive (isolates)	CR-negative (isolates)	Total (isolates)	CR rate (%)	OR (95% CI)	*P*
*Klebsiella pneumoniae*	DTR-positive	107	11	118	90.7	131.4 (56.3–306.5)	<0.001
	DTR-negative	41	555	596	6.9		
*Escherichia coli*	DTR-positive	16	20	36	44.4	26.6 (13.2–53.7)	<0.001
	DTR-negative	65	2164	2229	2.9		

Odds ratio (OR) represents the risk ratio of CR in the DTR-positive group relative to the DTR-negative group; CI, confidence interval. P values were calculated using the chi-square test or Fisher’s exact test.j

### 3.3 Longitudinal trends of carbapenem-resistant Enterobacterales from 2017 to 2025

Between 2017 and 2025, carbapenem resistance trajectories among Enterobacterales exhibited marked interspecies heterogeneity (**Figure 4**). Carbapenem-resistant *K. pneumoniae* (CRKP) remained at persistently high levels with pronounced annual fluctuations. Carbapenem-resistant *E. coli* (CRECO) remained at a consistently low baseline with only a slight upward drift. Carbapenem resistance in *Enterobacter cloacae* fluctuated annually between 3.6% and 15.8%.

**Figure 4:**
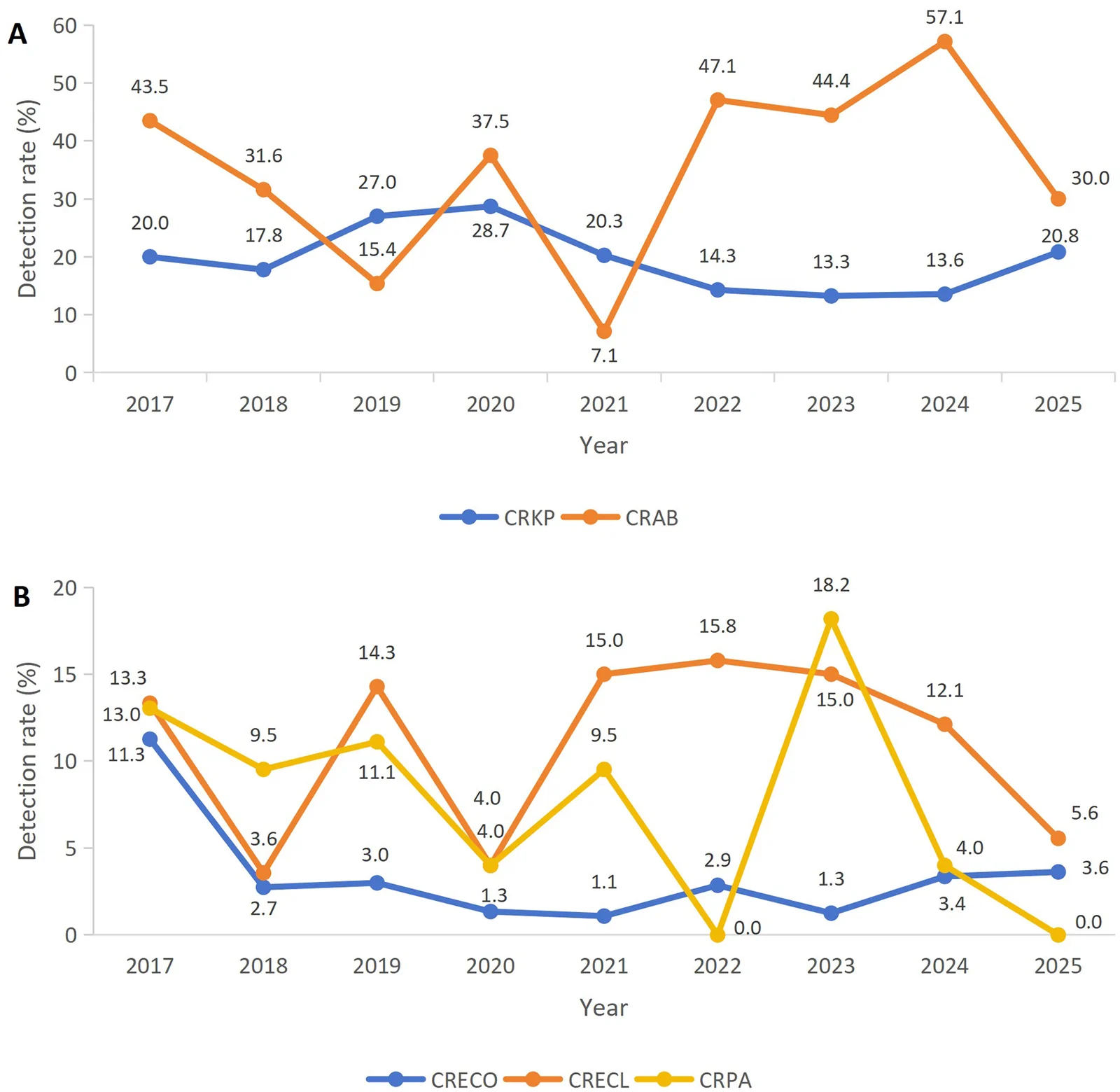
Annual trends in detection rates of carbapenem-resistant pathogens from 2017 to 2025. **(A)** Yearly detection trends of carbapenem-resistant *Klebsiella pneumoniae* (CRKP) and carbapenem-resistant *Acinetobacter baumannii* (CRAB). **(B)** Annual detection curves of carbapenem-resistant *Escherichia coli* (CRECO), carbapenem-resistant *Enterobacter cloacae* (CRECL), and carbapenem-resistant *Pseudomonas aeruginosa* (CRPA). CRKP, carbapenem-resistant *Klebsiella pneumoniae*; CRAB, carbapenem-resistant *Acinetobacter baumannii*; CRECO, carbapenem-resistant *Escherichia coli*; CRECL, carbapenem-resistant *Enterobacter cloacae;* CRPA, carbapenem-resistant *Pseudomonas aeruginosa*.

Carbapenem resistance in *Pseudomonas aeruginosa* remained generally low, with no resistant isolates detected in 2022 and 2025. In contrast, *A. baumannii* exhibited substantial interannual variability, reaching its peak in 2024.

Age-stratified analysis revealed significant differences in carbapenem resistance prevalence (**Figure 5**). For *E. coli*, resistance rates were significantly higher in older children than in neonates. For *P. aeruginosa*, neonates exhibited higher resistance rates. No statistically significant age-related differences were observed for *K. pneumoniae*, *A. baumannii*, or *Enterobacter cloacae*. Full annual stratified resistance data are provided in **Supplementary Table 1**; **Table 2**.

**Figure 5:**
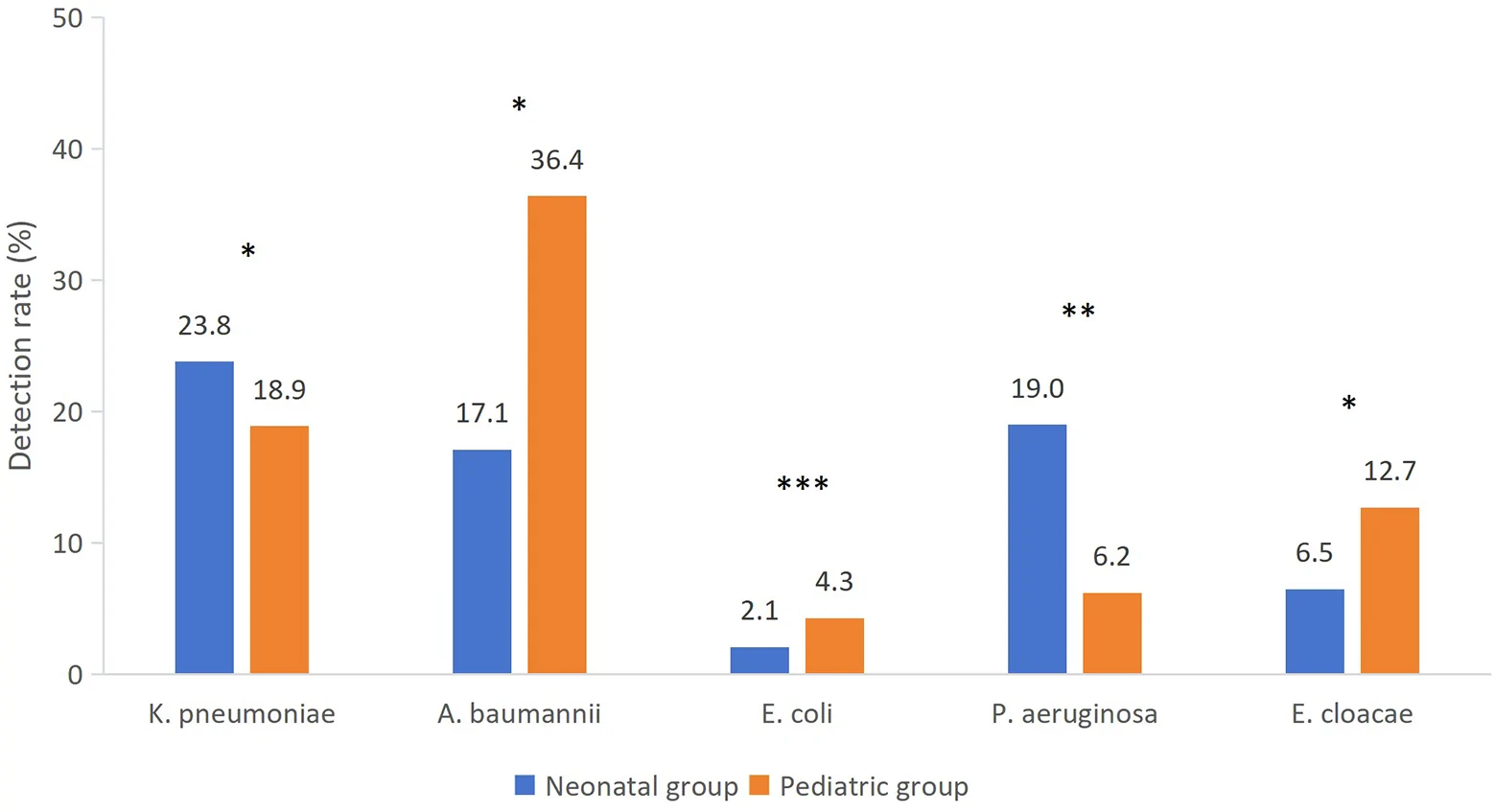
Comparison of detection rates of carbapenem-resistant pathogens in neonatal and pediatric groups. The neonatal subgroup included patients aged ≤ 28 days, and the pediatric subgroup covered patients aged 29 days to 14 years. Statistical significance: ***P < 0.01, **P < 0.05; *P ≥ 0.05.

### 3.4 Annual trends of vancomycin and linezolid MICs among *Staphylococcus aureus* over 9 years of surveillance

**Table 4** summarizes the overall antimicrobial resistance profiles of *S. aureus* across three pandemic stages. All collected *S. aureus* isolates remained fully susceptible to vancomycin and linezolid during the 9-year surveillance period. The vancomycin MIC distribution was consistent without temporal drift. Spearman correlation analysis revealed no significant annual trends in the proportion of isolates with MIC ≥2 μg/mL for both MRSA (*r* = 0.008, *P* = 0.980) and MSSA (*r*=−0.617, *P* = 0.077). No obvious time-dependent alterations were observed in vancomycin MIC_50_ and MIC_90_ of either strain (all *P* > 0.05). Detailed annual MIC_50_, MIC_90_ and full MIC distribution data are provided in **Supplementary Table 3**.

**Table 4:** Summary of antimicrobial resistance rates of *Staphylococcus aureus* during three COVID-19 pandemic phases.

Antimicrobial agent	2017–2019 (n=438)	2020–2022 (n=397)	2023–2025 (n=361)	*P*
Penicillin G	96.7	96.2	95.4	0.214
Oxacillin	32.9	34.3	29.1	0.028
Gentamicin	11.4	5.9	5.0	<0.001
Rifampicin	1.6	1.9	0.6	0.083
Ciprofloxacin	6.3	6.3	6.1	0.956
Levofloxacin	5.4	5.4	5.5	0.982
Trimethoprim-sulfamethoxazole	16.9	6.1	7.6	<0.001
Clindamycin	40.9	35.3	26.3	<0.001
Erythromycin	68.1	60.7	59.0	<0.001
Vancomycin	0.0	0.0	0.0	—
Linezolid	0.0	0.0	0.0	—

n, number of isolates. P values were calculated using the chi-square test for overall comparison across the three pandemic stages (pre-pandemic, pandemic, post-pandemic).

In contrast, an evident rightward shift of linezolid MIC was observed (**Figure 6**). The proportion of MRSA isolates with MIC ≥2 μg/mL increased significantly over years (*r* = 0.767, *P* = 0.016), whereas only a marginal upward trend was detected in MSSA isolates (*r* = 0.646, *P* = 0.058). Linezolid MIC_50_ remained steady at 2 μg/mL in both MRSA and MSSA throughout surveillance; the MIC_90_ of MRSA rose from 2 μg/mL to 4 μg/mL in 2025, while no significant temporal trends were found for linezolid MIC_50_ and MSSA MIC_90_ (all *P* > 0.05).

**Figure 6:**
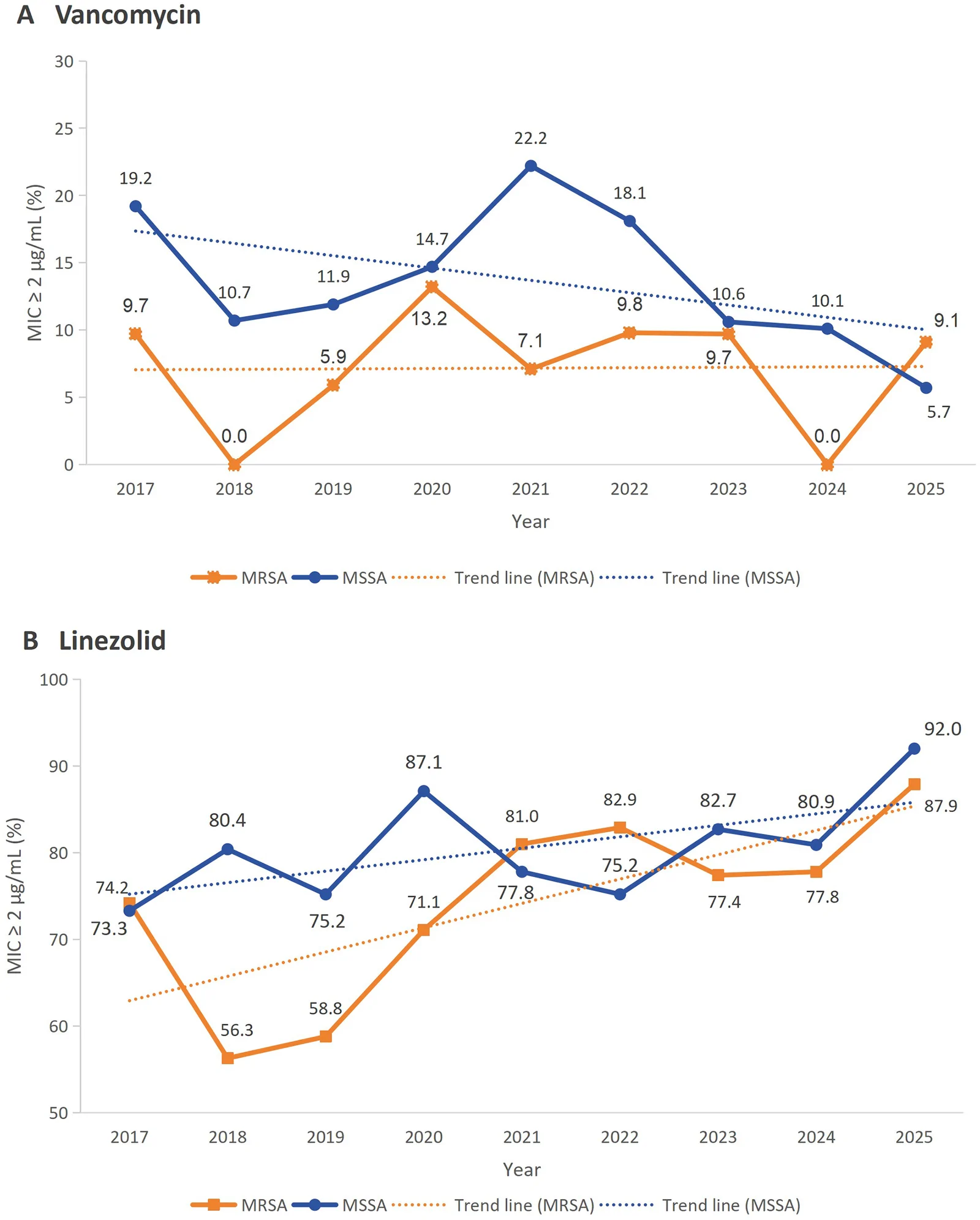
Curves showing annual proportions of Staphylococcus aureus isolates with MIC ≥2 μg/mL to vancomycin **(A)** and linezolid **(B)** from 2017 to 2025. MRSA, methicillin-resistant *Staphylococcus aureus*; MSSA, methicillin-susceptible *Staphylococcus aureus*. Dotted lines represent linear trends from two-tailed Spearman’s rank correlation test. Linezolid, MRSA: *r*= 0.767, *P* = 0.016; MSSA: *r* = 0.646, *P* = 0.058.

### 3.5 Antimicrobial susceptibility profile of *Enterococcus faecium*

A total of 515 *Enterococcus faecium* isolates were recovered in this study. The organism exhibited uniformly high susceptibility to vancomycin and linezolid, while maintaining persistently high resistance to ampicillin. Resistance to high-level gentamicin and fluoroquinolones showed modest fluctuations across pandemic phases.

As *E. faecium* was not a primary focus of the present study, detailed stratified resistance data are provided in **Supplementary Table 4**, with only a concise summary presented in the main text.

## 4 Discussion

### 4.1 Epidemiological significance of ESKAPEEc distribution and age-stratified characteristics

Based on nine years of continuous surveillance data from 134 medical institutions across Sichuan Province, we characterized the epidemiology of pediatric BSI pathogens within the ESKAPEEc group across the full COVID-19 era. The overall proportion of ESKAPEEc isolates was comparable to pediatric surveillance data reported from Beijing (Lyu et al., 2023), but lower than in adult cohorts (De Angelis et al., 2018). This likely reflects the distinct pediatric pathogen spectrum, in which coagulase-negative staphylococci predominate in early life and dilute the relative burden of resistant organisms, underscoring the limited transferability of adult resistance data to pediatric settings.

Temporal analysis demonstrated a characteristic pattern of increased ESKAPEEc detection during the pandemic period followed by a decline in the post-pandemic phase, reflecting the bidirectional regulatory impact of COVID-19 on nosocomial pathogen ecology (Li et al., 2025). *Escherichia coli* remained the leading ESKAPEEc pathogen in pediatric BSI, consistent with previous national and international reports (Crichton et al., 2018; Sajedi Moghaddam et al., 2024). Notably, pronounced age-stratified differences were identified as a key baseline finding. Neonates exhibited a higher risk of *Klebsiella pneumoniae* infection, whereas *Staphylococcus aureus* became the predominant pathogen in children older than three years. These patterns are closely associated with age-dependent immune maturation, exposure to invasive procedures, and the relative contributions of community- versus hospital-acquired infections. From a clinical standpoint, these data support stratified empiric therapy: neonatal severe infections should prioritize coverage of Gram-negative organisms such as *K. pneumoniae*, whereas regimens for older children should concurrently consider *S. aureus* coverage.

Moreover, the vast territory of Sichuan Province is characterized by marked regional heterogeneity in healthcare delivery. Tertiary hospitals predominantly manage critically ill children with invasive procedures and greater exposure to high-level antibiotics, whereas secondary hospitals treat milder cases but exhibit wide variability in microbiological testing capacity and infection control infrastructure. Such inter-institutional heterogeneity in patient case mix and preventive practices may confound the provincial estimates of bloodstream infection pathogen spectra and resistance rates, necessitating caution in the interpretation of our aggregate surveillance data. As COVID-19 gradually transitions to an endemic common infection, the epidemiological fluctuations of the ESKAPEEc group should not be attributed solely to pandemic-related factors. Changes in the disease spectrum and shifts in clinical practice patterns may also exert an impact on the prevailing trends of these pathogens.

### 4.2 Carbapenem resistance evolution and post-pandemic residual effects

Carbapenems remain a cornerstone therapy for severe pediatric Gram-negative BSI, and temporal changes in carbapenem resistance directly reflect the effectiveness of antimicrobial stewardship and infection control strategies (Sakagianni et al., 2025). In this study, carbapenem resistance in *K. pneumoniae* in Sichuan Province was at an intermediate national level—lower than reports from Beijing (Lyu et al., 2023) but higher than certain regional data from Southwest China (Yang et al., 2019). Carbapenem-resistant *E. coli* remained at a low endemic level, consistent with findings from Jiangxi Province (Zhou et al., 2023). In comparison with other domestic multicenter pediatric surveillance studies, the post-pandemic increase in carbapenem resistance among Klebsiella pneumoniae in our region was notably more pronounced, reflecting the distinctive epidemiological characteristics of antimicrobial resistance unique to the pediatric population in Sichuan. These regional discrepancies likely arise from differences in antimicrobial prescribing practices and the dissemination capacity of local resistant clones (González-Espinosa et al., 2026).

A key temporal finding was the significant increase in carbapenem resistance in both *E. coli* and *K. pneumoniae* during the post-pandemic period compared with the pre-pandemic baseline. Importantly, resistance levels did not return to baseline after relaxation of COVID-19 control measures, indicating a persistent post-pandemic legacy effect. During the pandemic, increased rates of severe bacterial co-infections and intensified carbapenem use exerted sustained selective pressure, promoting colonization and dissemination of resistant strains (Echegorry et al., 2024). Concurrently, healthcare system strain and reduced adherence to infection control measures further facilitated nosocomial transmission (Wee et al., 2021). Once stable endemic transmission chains are established within wards, restoration of routine infection control alone is insufficient to rapidly reverse resistance trends, highlighting the need for sustained carbapenem stewardship in the post-pandemic era.

Marked species-specific differences in carbapenem resistance were observed. *Pseudomonas aeruginosa* demonstrated limited capacity for sustained acquired resistance dissemination, resulting in overall low resistance levels. In contrast, *Acinetobacter baumannii*, owing to its strong environmental resilience, biofilm-forming capacity, and efficient horizontal gene transfer systems, persisted in hospital environments and represented a major challenge for pediatric infection control (Yamada et al., 2025). Age-stratified analysis further revealed higher *E. coli* carbapenem resistance in older children, whereas neonates exhibited increased susceptibility to carbapenem-resistant *P. aeruginosa*, providing important guidance for ward-level targeted surveillance strategies.

### 4.3 Clinical value of the DTR phenotype in surveillance

This study represents the first systematic epidemiological evaluation of DTR in a pediatric population in Southwest China, constituting a major conceptual innovation. Traditional MDR and XDR definitions rely on the number of resistant drug classes and fail to accurately capture scenarios with no viable therapeutic options. In contrast, the DTR framework more closely reflects real-world clinical treatment constraints in severe infections (Cosentino et al., 2023). However, pediatric epidemiological data on DTR in this region have been absent to date.

Substantial interspecies variability in DTR prevalence was observed among Gram-negative pathogens. *Acinetobacter baumannii* demonstrated an extremely high DTR positivity rate (87.3%), representing the most therapeutically intractable pathogen in pediatric BSI. DTR *A. baumannii* infections often necessitate agents such as polymyxins or tigecycline; however, pediatric indications and safety evidence for these agents remain limited, severely restricting therapeutic options (Meng et al., 2023; Dermitzaki et al., 2025).

Temporal trends showed a declining DTR rate in *A. baumannii* but a continuous linear increase in *K. pneumoniae*, suggesting species-specific differences in selective pressure and adaptation dynamics. This divergence supports the need for pathogen-specific dynamic early-warning systems rather than uniform surveillance approaches. Relative to other pediatric studies reported domestically, our findings revealed a remarkably high DTR prevalence in Acinetobacter baumannii, which constitutes a notable regional characteristic of bloodstream infections affecting children in Southwest China.

A critical finding from the cross-analysis of DTR and carbapenem resistance is that more than half of DTR-positive *E. coli* isolates were not carbapenem-resistant, indicating that DTR can arise independently through combined resistance to fluoroquinolones and broad-spectrum β-lactams. Conversely, most DTR *K. pneumoniae* isolates were concurrently carbapenem-resistant (Ohyama et al., 2026). These results demonstrate that reliance solely on carbapenem resistance would substantially underestimate the burden of difficult-to-treat *E. coli* infections. Therefore, incorporation of DTR surveillance into the Sichuan provincial resistance monitoring network is strongly recommended to enhance early warning capacity for pediatric severe infections.

Of note, the absence of individual patient clinical outcome data in this study prevented direct confirmation of a causal association between DTR positivity and mortality or treatment failure in our cohort. However, per the DTR definition and IDSA treatment guidelines (Kadri et al., 2018; Tamma et al., 2022), the DTR phenotype markedly limits therapeutic options in children, and the limited safety data for certain backup agents in this population underscore its value as a critical clinical risk alert. This study furnishes only a population-level epidemiological baseline; prospective cohort studies with clinical endpoints are thus required to further validate the prognostic utility of these markers.

### 4.4 Clinical warning implications of linezolid MIC drift in *S. aureus*

Systematic evaluation of linezolid MIC drift in pediatric populations in Southwest China has not been previously reported, making this a key contribution of the present study. Vancomycin and linezolid remain last-line agents for severe Gram-positive bloodstream infections in children, and continuous MIC monitoring is essential for preventing resistance emergence (Jian et al., 2020; Haas et al., 2023).

Over nine years, all *Staphylococcus aureus* isolates remained 100% susceptible to vancomycin and linezolid, with no resistant strains detected. However, their MIC trajectories differed substantially. Vancomycin MIC_50_, MIC_90_, and high-MIC subpopulations remained stable, consistent with previous pediatric surveillance data (Chen et al., 2023; Haas et al., 2023).

In contrast, a significant increase in the proportion of MRSA isolates with linezolid MIC ≥2 μg/mL was observed, with MSSA showing a borderline upward trend. By 2025, the MIC_90_ for MRSA reached 4 μg/mL, indicating early-stage linezolid MIC drift in local pediatric isolates. MIC creep reflects adaptive resistance: although isolates remain formally susceptible *in vitro*, antimicrobial efficacy gradually diminishes *in vivo*. Continued high-level exposure may further enrich high-MIC subpopulations, ultimately leading to clinically resistant strains (Han et al., 2025). Increased linezolid utilization during the pandemic, driven by higher rates of severe infections, likely served as a major selective pressure (Rawson et al., 2020), particularly in neonatal and pediatric intensive care units.

This MIC drift phenomenon has important regional public health implications. Clinically, strict indications for linezolid use should be enforced to avoid unnecessary empirical administration. At the laboratory level, annual MIC stratification surveillance systems should be established, with particular attention to MRSA MIC distribution shifts to enable early intervention. The observed post-pandemic decline in overall MRSA detection further supports the effectiveness of standard infection control measures, including isolation precautions and hand hygiene, in limiting transmission (Wee et al., 2021).

### 4.5 Bidirectional impact of COVID-19 on resistance ecology

Integrated temporal analysis indicates that the COVID-19 pandemic exerted a sustained bidirectional influence on pediatric BSI pathogen distribution and resistance patterns. On one hand, strict infection control measures—including isolation protocols, hand hygiene reinforcement, and environmental disinfection—reduced transmission of common organisms and contributed to modest reductions in resistance to macrolides and aminoglycosides (Wang and Li, 2022). On the other hand, increased severity of infections and intensified use of broad-spectrum antimicrobials continuously selected for multidrug-resistant organisms, driving the rise of carbapenem resistance and linezolid MIC drift (Rawson et al., 2020; Thomas et al., 2022).

Importantly, infection control interventions primarily reduce transmission efficiency but do not eliminate antibiotic-driven selective pressure. Consequently, resistance prevalence did not decline after relaxation of pandemic measures, resulting in a persistent post-pandemic legacy effect. These findings emphasize that antimicrobial stewardship and infection prevention must be maintained in parallel over the long term, rather than being relaxed following the resolution of public health emergencies.

*Enterococcus faecium*, as a secondary Gram-positive pathogen, demonstrated stable susceptibility profiles, with persistently high resistance only to penicillin and ampicillin, and maintained susceptibility to vancomycin and linezolid. Its resistance pattern was largely unaffected by the pandemic, supporting continuation of existing treatment strategies in routine clinical practice.

### 4.6 Control recommendations, limitations, and future directions

Based on these findings, several stratified recommendations are proposed for pediatric BSI resistance control in Sichuan Province: (1) neonatal wards should prioritize prevention of carbapenem-resistant *K. pneumoniae* and *P. aeruginosa*, with strengthened aseptic management of invasive procedures; (2) pediatric ICUs should strictly regulate carbapenem and linezolid use and establish annual MIC monitoring systems; (3) regional surveillance networks should incorporate DTR as a routine indicator to enable early warning of difficult-to-treat infections; and (4) age-stratified empiric therapy guidelines should be developed to reduce unnecessary broad-spectrum antibiotic exposure.

This study has several limitations. First, as a single-region retrospective surveillance study, generalizability to other regions is limited. Second, although all participating laboratories adhered to CLSI M100 (35th edition) standards (Clinical and Laboratory Standards Institute, 2025), variability across 134 institutions may introduce minor measurement bias. Third, clinical outcome data such as hospitalization duration, treatment regimens, and prognosis were not available, limiting assessment of the prognostic impact of DTR and high-MIC strains. Fourth, this study relied solely on phenotypic susceptibility testing to define carbapenem-resistant (CR), difficult-to-treat resistance (DTR), and linezolid MIC creep. Owing to limitations of the surveillance network, no bacterial isolates were available for carbapenemase gene detection or whole-genome sequencing. Consequently, this study reflects only phenotypic epidemiological trends over time and is unable to distinguish whether resistance arose from horizontal transfer of resistance genes or from nosocomial spread of dominant clones. Furthermore, it precludes elucidation of the molecular genetic backgrounds underlying the DTR phenotype and the linezolid high-MIC subpopulations, thereby restricting our capacity for strain transmission tracing and source attribution. Fifth, Subgroup analyses according to hospital tier (tertiary versus secondary hospitals) were not performed to evaluate differences in antimicrobial resistance profiles. For certain bacterial species, the number of isolates recovered from secondary hospitals was limited, resulting in insufficient sample sizes for subgroup stratification, which may compromise the statistical robustness of the findings. Consequently, this study was unable to delineate the distinct epidemiological characteristics of antimicrobial resistance between different levels of healthcare facilities. Sixth, annual and institutional data on pediatric antimicrobial consumption were unavailable in this study, precluding quantitative analysis of the association between the intensity of carbapenem and linezolid use and the temporal changes in resistance. Therefore, inferences regarding antimicrobial selective pressure were drawn solely from epidemiological temporal trends and previously published literature, without direct evidence of local antibiotic exposure. This constitutes an important confounding factor in the present study.

Future work will address these limitations through: (1) whole-genome sequencing of DTR isolates and high-MIC *S. aureus* strains to elucidate resistance mechanisms and clonal relationships; (2) prospective cohort studies incorporating clinical outcomes to quantify prognostic impacts; (3) stratified analyses by hospital level to refine regional control strategies; and (4) integration of antimicrobial consumption data to quantitatively assess the relationship between drug exposure and resistance evolution.

## 5 Conclusions

Based on nine years of continuous pediatric bloodstream infection surveillance across 134 hospitals in Sichuan Province, this study delineates the full-spectrum epidemiology of ESKAPEEc pathogens across the COVID-19 era and provides integrated insights into carbapenem resistance, DTR, and linezolid MIC evolution in children.

Notably, ESKAPEEc detection exhibited a transient increase during the pandemic followed by a post-pandemic decline, with pronounced age-dependent pathogen stratification. The COVID-19 pandemic was associated with a sustained increase in carbapenem resistance among *Escherichia coli* and *Klebsiella pneumoniae*, and this resistance burden demonstrated a persistent post-pandemic legacy effect. Moreover, distinct Gram-negative bacteria exhibited heterogeneous DTR epidemiology. *Acinetobacter baumannii* showed an extremely high overall DTR burden but demonstrated a declining trend over time, whereas DTR in *K. pneumoniae* increased in a continuous linear manner. Reliance on carbapenem resistance alone would fail to identify approximately half of DTR-positive *E. coli* isolates. These findings highlight the necessity of incorporating DTR surveillance into regional antimicrobial resistance monitoring systems. In addition, within the local pediatric population, vancomycin MIC values of *Staphylococcus aureus* remained stable without evidence of drift; however, linezolid MIC values in MRSA isolates showed a significant upward trend, indicating a potential long-term risk of emerging resistance. Accordingly, linezolid use in neonatal and pediatric intensive care units should be strictly regulated, and annual MIC-based dynamic surveillance should be implemented.

Collectively, COVID-19 has exerted a sustained and bidirectional impact on pediatric resistance ecology in Sichuan Province. Carbapenem resistance expansion, high DTR burden in A. baumannii, and early linezolid MIC drift in MRSA represent the major ongoing challenges. These findings support age-stratified empirical therapy, routine incorporation of DTR and MIC surveillance, and sustained antimicrobial stewardship combined with infection control to mitigate further dissemination of multidrug-resistant organisms.

## Data Availability

The datasets presented in this study can be found in online repositories. The names of the repository/repositories and accession number(s) can be found in the article/**Supplementary Material**.
