# Supplementary material for: Distribution, carbapenem resistance and DTR dynamics of ESKAPEEc pathogens from pediatric bloodstream infections in Sichuan Province, 2017-2025: epidemiological characteristics under COVID-19 pandemic and emerging linezolid MIC creep
Source: Front Cell Infect Microbiol. 2026 Oct 1;16:1914448. doi: 10.3389/fcimb.2026.1914448 (PMC13668538; doi:10.3389/fcimb.2026.1914448)
Supplement: Supplementary file 1 [file Table1.docx]

Table 1 Annual stratified raw data of carbapenem resistance for each bacterial species, 2017–2025

| **Bacterial species** | **2017** | **2018** | **2019** | **2020** | **2021** | **2022** | **2023** | **2024** | **2025** |
| --- | --- | --- | --- | --- | --- | --- | --- | --- | --- |
| CRKP | 20.0 | 17.8 | 27.0 | 28.7 | 20.3 | 14.3 | 13.3 | 13.6 | 20.8 |
| CRAB | 43.5 | 31.6 | 15.4 | 37.5 | 7.1 | 47.1 | 44.4 | 57.1 | 30.0 |
| CRECO | 11.3 | 2.7 | 3.0 | 1.3 | 1.1 | 2.9 | 1.3 | 3.4 | 3.6 |
| CRECL | 13.3 | 3.6 | 14.3 | 4.0 | 15.0 | 15.8 | 15.0 | 12.1 | 5.6 |
| CRPA | 13.0 | 9.5 | 11.1 | 4.0 | 9.5 | 0.0 | 18.2 | 4.0 | 0.0 |

Note: CRKP, carbapenem-resistant *Klebsiella pneumoniae*; CRAB, carbapenem-resistant *Acinetobacter baumannii*; CRECO, carbapenem-resistant *Escherichia coli*; CRECL, carbapenem-resistant *Enterobacter cloacae*; CRPA, carbapenem-resistant *Pseudomonas aeruginosa*. Only annual resistance proportions are presented; annual absolute counts of resistant/total isolates were not archived, so Cochran-Armitage trend test could not be performed. Spearman rank-order correlation was used as an alternative: CRKP *r*=−0.300, *P*=0.433; CRAB *r*=0.267, *P*=0.488; CRECO *r*=0.008, *P*=0.983; CRECL *r*=0.184, *P*=0.635; CRPA *r*=−0.506, *P*=0.164. No significant temporal trend was observed for any species.

Table 2 Stratified carbapenem resistance detection rates for neonates and children, 2017–2025

| **Bacterial species** | **Neonates(≤28d)** | | | **Children** **(29d–<14y)** | | |
| --- | --- | --- | --- | --- | --- | --- |
|  | **Detected isolates** | **Total isolates** | **Detection rate (%)** | **Detected isolates** | **Total isolates** | **Detection rate (%)** |
| CRECO | 16 | 755 | 2.1 | 65 | 1510 | 4.3 |
| CRKP | 64 | 269 | 23.8 | 84 | 445 | 18.9 |
| CRECL | 4 | 62 | 6.5 | 20 | 157 | 12.7 |
| CRAB | 6 | 35 | 17.1 | 39 | 107 | 36.4 |
| CRPA | 4 | 21 | 19.0 | 9 | 146 | 6.2 |

Note: CRKP, carbapenem-resistant *Klebsiella pneumoniae*; CRAB, carbapenem-resistant *Acinetobacter baumannii*; CRECO, carbapenem-resistant *Escherichia coli*; CRECL, carbapenem-resistant *Enterobacter cloacae*; CRPA, carbapenem-resistant *Pseudomonas aeruginosa*. This table presents pooled age‑stratified data across the whole study period rather than annual time‑series data; therefore Cochran‑Armitage trend test was not performed.

Table 3 Annual MIC₅₀, MIC₉₀ and concentration distribution of vancomycin and linezolid in Staphylococcus aureus, 2017–2025

| **Year** | **Strain type** | **Isolates** | **Vancomycin MIC distribution (μg/mL)** | | | | | |  | **Linezolid MIC distribution (μg/mL)** | | | | | |
| --- | --- | --- | --- | --- | --- | --- | --- | --- | --- | --- | --- | --- | --- | --- | --- |
|  |  |  | **≤0.5** | **1** | **≥2** | **≥2 （%）** | **MIC_50_** | **MIC_90_** |  | **≤0.5** | **1** | **≥2** | **≥2 （%）** | **MIC_50_** | **MIC_90_** |
| 2017 | MRSA | 31 | 10 | 18 | 3 | 9.7 | 1 | 2 |  | 2 | 6 | 23 | 74.2 | 2 | 2 |
|  | MSSA | 120 | 15 | 82 | 23 | 19.2 | 1 | 2 |  | 5 | 27 | 88 | 73.3 | 2 | 2 |
| 2018 | MRSA | 32 | 17 | 15 | 0 | 0.0 | 0.5 | 2 |  | 3 | 11 | 18 | 56.3 | 2 | 2 |
|  | MSSA | 112 | 12 | 88 | 12 | 10.7 | 1 | 2 |  | 4 | 18 | 90 | 80.4 | 2 | 2 |
| 2019 | MRSA | 34 | 11 | 21 | 2 | 5.9 | 1 | 2 |  | 2 | 12 | 20 | 58.8 | 2 | 2 |
|  | MSSA | 109 | 10 | 86 | 13 | 11.9 | 1 | 2 |  | 3 | 24 | 82 | 75.2 | 2 | 2 |
| 2020 | MRSA | 38 | 12 | 21 | 5 | 13.2 | 1 | 2 |  | 2 | 9 | 27 | 71.1 | 2 | 2 |
|  | MSSA | 116 | 14 | 85 | 17 | 14.7 | 1 | 2 |  | 3 | 12 | 101 | 87.1 | 2 | 2 |
| 2021 | MRSA | 42 | 15 | 24 | 3 | 7.1 | 1 | 2 |  | 2 | 6 | 34 | 81.0 | 2 | 2 |
|  | MSSA | 99 | 11 | 66 | 22 | 22.2 | 1 | 2 |  | 4 | 18 | 77 | 77.8 | 2 | 2 |
| 2022 | MRSA | 41 | 14 | 23 | 4 | 9.8 | 1 | 2 |  | 2 | 5 | 34 | 82.9 | 2 | 2 |
|  | MSSA | 105 | 12 | 74 | 19 | 18.1 | 1 | 2 |  | 3 | 23 | 79 | 75.2 | 2 | 2 |
| 2023 | MRSA | 31 | 10 | 18 | 3 | 9.7 | 1 | 2 |  | 2 | 5 | 24 | 77.4 | 2 | 2 |
|  | MSSA | 104 | 13 | 80 | 11 | 10.6 | 1 | 2 |  | 4 | 14 | 86 | 82.7 | 2 | 2 |
| 2024 | MRSA | 18 | 9 | 9 | 0 | 0.0 | 0.5 | 1 |  | 1 | 3 | 14 | 77.8 | 2 | 2 |
|  | MSSA | 89 | 11 | 69 | 9 | 10.1 | 1 | 2 |  | 3 | 14 | 72 | 80.9 | 2 | 2 |
| 2025 | MRSA | 33 | 11 | 19 | 3 | 9.1 | 1 | 2 |  | 1 | 3 | 29 | 87.9 | 2 | 4 |
|  | MSSA | 87 | 12 | 70 | 5 | 5.7 | 1 | 2 |  | 2 | 5 | 80 | 92.0 | 2 | 2 |

Note: MRSA, methicillin-resistant Staphylococcus aureus; MSSA, methicillin-susceptible *Staphylococcus aureus*; MIC, minimum inhibitory concentration. Susceptibility results were interpreted per CLSI M100 35th edition. Spearman’s rank‑order correlation (two‑tailed) was used to analyze annual trends of MIC ≥2 μg/mL proportion:

Vancomycin, MRSA: *r*=0.008, *P*=0.980

Vancomycin, MSSA: *r*=−0.617, *P*=0.077

Linezolid, MRSA: *r*=0.767, *P*=0.016

Linezolid, MSSA: *r*=0.646, *P*=0.058

Table 4 Stratified antimicrobial susceptibility data of Enterococcus faecium in three pandemic phases

| **Antimicrobial agent** | **Pre-pandemic period (2017–2019)** | **Pandemic period (2020–2022)** | **Post-pandemic period (2023–2025)** |
| --- | --- | --- | --- |
| Penicillin G | 85.8 | 90.8 | 93.2 |
| Ampicillin | 86.3 | 89.7 | 91.6 |
| High-level gentamicin | 37.8 | 48.5 | 42.6 |
| Ciprofloxacin | 70.6 | 56.6 | 70.7 |
| Levofloxacin | 54.1 | 46.2 | 53.7 |
| Erythromycin | 75.4 | 53.5 | 56.5 |
| Vancomycin | 0.0 | 1.4 | 0.6 |
| Linezolid | 0.0 | 0.0 | 0.0 |

Note: Data are presented as resistance rate (%).This table compares resistance rates across three pre‑defined pandemic phases instead of continuous yearly observations; Cochran‑Armitage trend test was not applicable.

Table 5 Complete Annual Antimicrobial Resistance Rates of All ESKAPEEc Isolates

| **Pathogen** | **Period** | **TZP** | **CAZ** | **CTX** | **FEP** | **IPM** | **MEM** | **AMK** | **GEN** | **CIP** | **LVX** | **ATM** |
| --- | --- | --- | --- | --- | --- | --- | --- | --- | --- | --- | --- | --- |
| *E. coli* | 2017-2019 | 4.2 | 15.6 | 42.0 | 16.3 | 2.1 | 3.2 | 1.2 | 31.9 | 32.2 | 27.4 | 23.0 |
|  | 2020-2022 | 4.8 | 15.8 | 46.3 | 13.7 | 1.5 | 2.5 | 2.5 | 30.0 | 40.2 | 32.3 | 25.1 |
|  | 2023-2025 | 5.9 | 15.7 | 40.3 | 16.9 | 2.8 | 2.9 | 0.5 | 25.7 | 36.8 | 32.1 | 23.7 |
| *K. pneumoniae* | 2017-2019 | 29.5 | 44.5 | 61.6 | 37.6 | 18.3 | 23.4 | 1.4 | 16.5 | 25.2 | 8.1 | 41.7 |
|  | 2020-2022 | 30.8 | 36.5 | 55.9 | 31.2 | 22.5 | 28.7 | 3.7 | 17.4 | 32.3 | 17.2 | 39.1 |
|  | 2023-2025 | 28.0 | 44.7 | 56.9 | 38.5 | 15.2 | 15.2 | 2.5 | 16.0 | 32.7 | 15.4 | 44.4 |
| *P. aeruginosa* | 2017-2019 | 14.8 | 14.5 | — | 14.5 | 11.3 | 9.5 | — | 1.6 | 4.8 | 1.7 | 34.4 |
|  | 2020-2022 | 3.8 | 9.6 | — | 5.7 | 3.8 | 3.4 | — | 2.3 | 3.8 | 6.1 | 17.9 |
|  | 2023-2025 | 3.8 | 3.8 | — | 1.9 | 5.9 | 6.1 | — | 2.5 | 1.9 | 6.0 | 2.9 |
| *A. baumanni* | 2017-2019 | 26.2 | 29.3 | 48.0 | 30.8 | 28.9 | 40.5 | 23.2 | 27.5 | 30.4 | 23.7 | 44.1 |
|  | 2020-2022 | 25.8 | 33.3 | 32.0 | 32.4 | 32.4 | 30.0 | 23.1 | 31.2 | 26.5 | 27.3 | 54.8 |
|  | 2023-2025 | 35.3 | 42.3 | 35.7 | 42.3 | 43.5 | 40.0 | 33.3 | 34.6 | 44.0 | 29.2 | 47.8 |

Note: Data are presented as resistance rate (%).Abbreviations: *E. coli, Escherichia coli; K. pneumoniae, Klebsiella pneumoniae; P. aeruginosa, Pseudomonas aeruginosa; A. baumanni, Acinetobacter baumannii*. TZP, piperacillin-tazobactam; CAZ, ceftazidime; CTX, cefotaxime; FEP, cefepime; IPM, imipenem; MEM, meropenem; AMK, amikacin; GEN, gentamicin; CIP, ciprofloxacin; LVX, levofloxacin; ATM, aztreonam. The symbol "−" indicates that susceptibility testing was not conducted for the corresponding pathogen-antibiotic pair.
